# Chemokine Receptor Profile of T Cells and Progression Rate of Geographic Atrophy Secondary to Age-related Macular Degeneration

**DOI:** 10.1167/iovs.65.1.5

**Published:** 2024-01-02

**Authors:** Jenni Martinez Villarruel Hinnerskov, Marie Krogh Nielsen, Alexander Kai Thomsen, Maria Abildgaard Steffensen, Bent Honoré, Henrik Vorum, Mogens Holst Nissen, Torben Lykke Sørensen

**Affiliations:** 1 Department of Ophthalmology, Zealand University Hospital, Roskilde, Denmark; 2 Faculty of Health and Medical Sciences, University of Copenhagen, Copenhagen, Denmark; 3 Department of Immunology and Microbiology, University of Copenhagen, Copenhagen, Denmark; 4 Department of Biomedicine, Aarhus University, Aarhus, Denmark; 5 Department of Clinical Medicine, Aalborg University Hospital, Aalborg, Denmark; 6 Department of Ophthalmology, Aalborg University Hospital, Aalborg, Denmark

**Keywords:** age-related macular degeneration, geographic atrophy, chemokine receptor, single-nucleotide polymorphisms

## Abstract

**Purpose:**

Geographic atrophy (GA) secondary to age-related macular degeneration is a progressive retinal degenerative disease. Systemic chemokine receptors and known risk-associated single-nucleotide polymorphisms have been associated with GA pathogenesis. Because halting progression is pivotal for patients, we investigated the association of candidate chemokine receptors and progression rate (PR) of atrophic lesions in patients with GA.

**Methods:**

This prospective observational study conducted at a single center included 85 patients with GA and 45 healthy controls. Patients were followed up after 13 months on average. Serial fundus autofluorescence images were used to determine the PR of atrophic lesions. The proportion of chemokine receptors on peripheral lymphocytes were determined by flow cytometric analysis.

**Results:**

Patients with GA had a lower proportion of CCR6 on CD8+T cells compared to healthy controls. Importantly, the proportion of CCR6 on CD4+T cells was lower in patients with fast GA progression compared to patients with slow progression of disease, suggesting that dysregulation of CCR6 could be involved in progression of GA. We also found that GA patients had a markedly higher percentage of CCR5 on CD4+ and CD8+T cells compared to healthy controls. After stratification according to ARMS2 polymorphism, we found a significantly lower level of CCR5 on CD8+T cells among patients with high-risk genotypes compared with patients with the low-risk genotype.

**Conclusions:**

Our study finds that chemokine receptors are dysregulated in patients with GA and that CCR6 might be involved in GA progression, making it a potential target for intervention.

Age-related macular degeneration (AMD) is a multifactorial disease and the leading cause of visual impairment among elderly in developed countries. In the advanced end stage, AMD is subdivided into geographic atrophy (GA) and neovascular AMD (nAMD). GA represents a late stage of dry AMD characterized by ocular coherence tomography (OCT) imaging features of complete retinal pigment epithelial and outer retinal atrophy defined by the Classification of Atrophy Meeting Group.^1^ The pathogenesis of GA has been widely studied and has for many years remained largely unknown. However, strong emerging evidence points toward the involvement of the complement system in GA. The complement system, which forms part of the innate immune system, has been extensively studied in relation to GA, and its importance has been established with the recent approval from the Food and Drug Administration of two medical treatments for GA.^2^^,^^3^ The adaptive system could also play a role in the pathogenesis of GA. The presence of lymphocytes in eyes of GA was initially detected by Penfold et al.,^4^^,^^5^ and CD8+ cells have been found in the choroid of human donor eyes with advanced AMD with or without drusen.^6^ Furthermore, Faber et al.^7^ showed that the presence of peripheral CD56+ CD28− memory T cells in patients with all stages of AMD, including GA was associated with a 3.5-fold increased risk of developing AMD.

Accumulating evidence suggests that specific signal molecules, including chemokines, are associated with AMD. Chemokines are a subfamily of small size cytokines and are primarily known for their ability to stimulate the migration of leukocytes to sites of inflammation. Besides their chemotactic function, chemokines have a variety of other activities, exerting their effects via binding to chemokine receptors, which are located at target cell surfaces.

A major risk factor for AMD is aging. Further, the immune system including the chemokine receptor profile of T cells have shown to change with age. Specifically for chemokine receptors, it is known that aging affects the surface expression of certain chemokine receptors on T cells, whereas other chemokine receptors are increased. For instance, Mo et al.^8^ demonstrated that CD4+ T cells from aged mice had an increased expression of CCR1, 2, 4, 5, 6, 8, and CXCR2-5 and a decreased expression of CCR7 and 9 compared to younger mice. In humans, an increased expression of CCR5 on CD8+ T cells has been found in frail elderly individuals compared to non-frail.^9^ Additionally, Yung et al.^10^ showed that aging in humans was associated with an increase in CCR1-5 gene expression in CD4+ T cells.

Studies have reported imbalances of chemokines to be associated with the development of AMD, and specific chemokines and chemokine receptors have been shown to be involved in drusen formation and RPE changes seen in early AMD.^11^^–^^13^ However, these studies are carried out on murine models of AMD and studies investigating chemokines and chemokine receptors in patients with AMD, specifically GA, remain sparse. Our group and others have found alterations in expression levels of various chemokine receptors on T cells in AMD patients compared to healthy controls.^14^^–^^22^ The chemokine receptor CXCR3, which is expressed on T cells, B cells and natural killer (NK) cells,^23^ has been shown to hold antifibrotic and angiostatic abilities via binding to its ligand CXCL10.^24^^–^^26^

CCR6 is widely expressed on many different types of immune cells and has been associated with several autoimmune and inflammatory disorders.^27^ It binds exclusively to its chemokine ligand CCL20, unlike other chemokine receptors, which can be activated by more than one chemokine. Only a few studies have investigated CCR6 in relation to AMD,^14^^,^^21^ with our group being the only one to have specifically studied CCR6 expression levels in nonexudative AMD patients, including GA.^14^

Furthermore, our group has previously found an increased expression of the chemokine receptor CCR5 in GA patients, specifically. Its ligand CCL5, also known as RANTES (regulated on activation, normal T cell expressed and secreted) has been shown to be secreted by RPE cells^28^ and is suggested to be implicated in AMD pathogenesis.^18^^,^^19^^,^^22^^,^^29^ CCR5 and its ligand has also been linked to other neurodegenerative age-related diseases such as Alzheimer's and Parkinson disease.^30^^–^^33^

Other risk factors known to contribute to GA pathogenesis include smoking and genetic predisposition. The two main genetic variants, known as single nucleotide polymorphisms (SNP), in the complement factor H (CFH) and age-related maculopathy susceptibility 2 (ARMS2) genes have been strongly associated with the risk of developing AMD.^34^^–^^41^

Because the chemokine receptors CXCR3, CCR6, and CCR5 have previously been shown to be altered in observational studies,^14^^–^^22^ we sought to investigate the effect of alterations in T-cell chemokine receptor expression on progression rate (PR) of atrophic lesions. Furthermore, we explored whether these alterations differed according to known risk-associated SNPs (CFH rs1061170, ARMS2 rs10490924).

## Methods

### Participants and Inclusion

This prospective observational study was approved by the Ethics Committee, Region Zealand, Denmark (Reg. no. SJ-768) and adhered to the tenets of the Declaration of Helsinki. Before inclusion, participants had the nature and possible consequences of the study explained and oral and written informed consent was obtained from each participant.

We consecutively recruited participants from the Department of Ophthalmology of Zealand University Hospital, Roskilde, Denmark between October 2019 to July 2021. Patients with GA caused by AMD were recruited from the outpatient retinal program and for the control group, patient spouses, and patients attending the outpatient program were invited to participate. GA patients were eligible if they had no prior history or current diagnosis of nAMD. Individuals qualified to enter the control group if they were above 60 years of age and had no current or prior history of retinal disease. Each patient underwent follow-up examination after minimum seven months between August 2020 to June 2022.

Patients and control individuals were not included if they had active cancer, systemic inflammatory or infectious disease, use of immune modulating treatment, prior anti-VEGF therapy injections, and c-reactive protein (CRP) levels >16 mg/L. Current smokers were also excluded from the study, as tobacco triggers acute inflammation mediated via Toll-like receptors^42^ and modulates the expression of pro-inflammatory cytokines and chemokines.^43^

### Clinical Data and Image Acquisition

All participants were subjected to a structured interview regarding their medical history, current health status, medication, and lifestyle. Prior to retinal imaging, participants’ pupils were dilated using tropicamide 1%. Retinal imaging consisted of stereoscopic 45° color fundus photographs centered on the macula (model TRG-NW8; Topcon, Tokyo, Japan) and blue fundus autofluorescence (FAF) images obtained by using the Heidelberg Spectralis device (Heidelberg Engineering, Heidelberg, Germany) with a standard 30° field of view (768 × 768 pixels). The automatic real time mode was used averaging 23 images to form a mean image. Spectralis HRA+OCT (Heidelberg Engineering, Heidelberg, Germany) was used with the automatic real time mode to obtain 20° × 20°, 49 SD-OCT B-scans centered on the fovea.

### Image Analysis

Follow-up examination on all GA patients was performed after at least seven months from baseline. On each patient only one eye was used as the study eye. Consistently we chose the right eye; however, in case of unilateral GA in the left eye or poor image quality in the right eye, the left eye was included as the study eye.

Segmentation of the GA area was performed on blue FAF images using a semi-automated MATLAB-based software (MathWorks, Natick, MA, USA). The in-build MATLAB Image Processing Toolbox was applied in a script using the Image Segmenter specifically. In cases of multifocal GA lesions, lesions with an area above 0.05 mm² were selected and compiled as an image mask using an adaptive associating pixel marking tool to identify and select specific neighboring pixels with similar color and light properties. Constraints were manually put in place to exclude OCT image-verified normal foveal hypoautoflourescence, GA contiguous with the optic nerve or vessels not accurately detected by the Image Segmenter. The MATLAB script enabled an iterative workflow tailored for batch-processing of images. The image mask was applied to obtain a binary image. The GA area was calculated by counting all TRUE (value = 1) pixels within the binary image hereafter multiplying by the defined area per pixel. The applied method using MATLAB-based software is not typically used for measurements of GA areas. Hence, we performed a Bland-Altman analysis to evaluate the level of agreement between Heidelberg RegionFinder (gold standard) and the described MATLAB-script used for GA area measurements. The two methods for GA area measurement were compared on 50 randomly selected images. One investigator (J.V.H.) analyzed all FAF images by using the described MATLAB-based software, and another investigator (L.F.G.) analyzed 24 images by the same method to test intergrader agreement.

The PR was determined on each patient by subtracting the area of atrophy (mm²) at follow-up visit from the area of atrophy at baseline divided by the time of follow-up (in years). To determine PR normalized for baseline lesion size a square root transformation strategy was applied.^44^ After transformation, we calculated the standard cutoff points in order to divide our GA cohort in three, based on the upper and lower square root PR tertiles. The 33.33rd and 66.67th percentiles gave us the upper and lower tertiles, respectively. The cutoff points were 0.174 mm/y (lower tertile) and 0.306 mm/y (upper tertile). The lower tertile was defined as slow GA progressors and the upper tertile as fast GA progressors.

### Blood Sampling, Flow Cytometry, and Genotyping

Peripheral blood samples were drawn from antecubital veins from each participant at baseline visit. Ethylenediaminetetraacetic-acid–coated tubes were used for flow cytometry and SNP analysis. Tubes used for SNP analysis were immediately stored frozen at −80°C for later use. Lithium-heparin coated tubes were used for CRP analysis.

**Figure 1.**
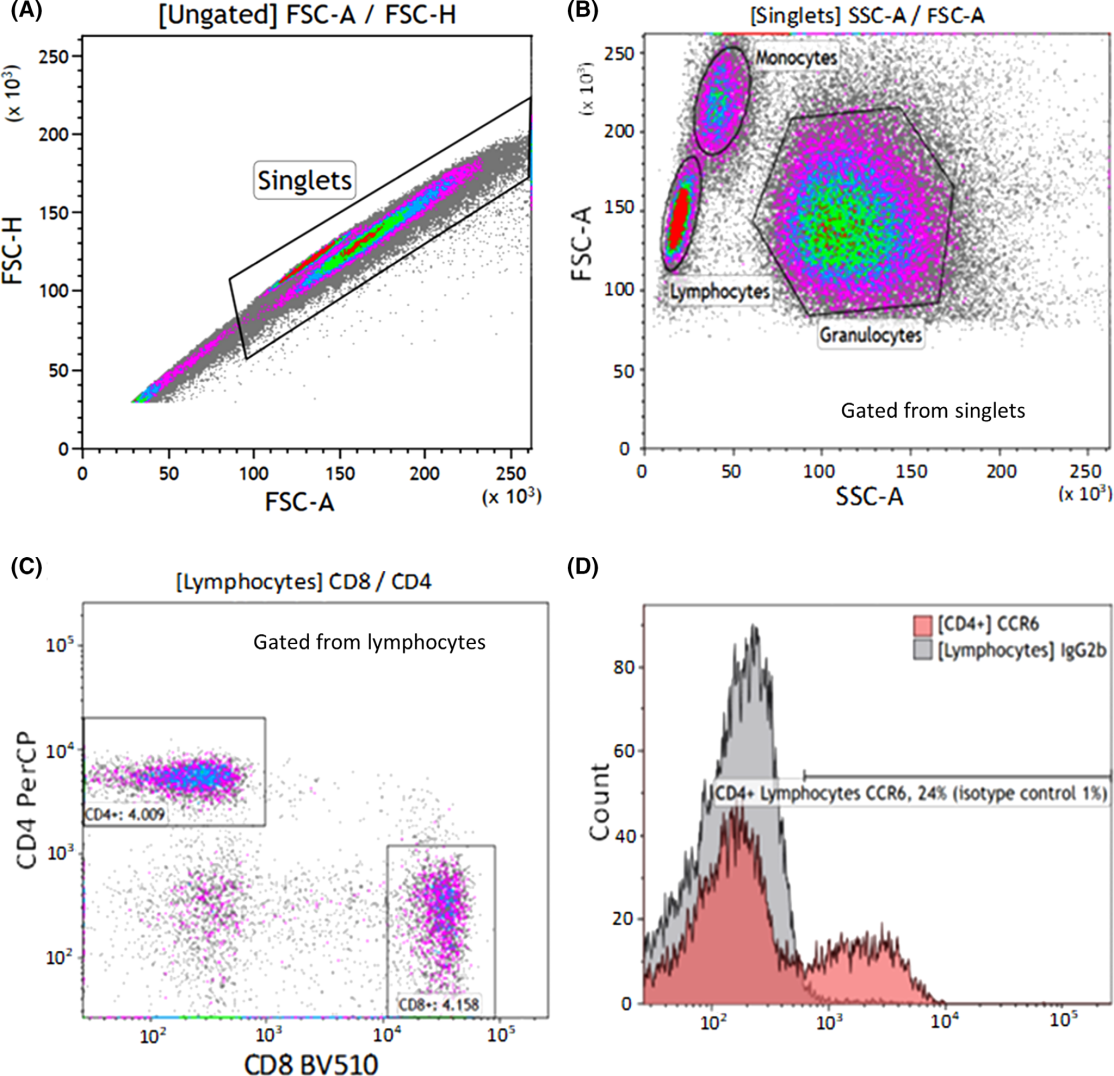
Gating strategy for identifying the proportion of chemokine receptor expression on lymphocytes using Kaluza software. (**A**) Singlets were identified on a forward scatter-height (FSC-H) versus forward scatter-area (FSC-A) plot. (**B**) Lymphocytes were identified on an FSC-A versus side-scatter-area (SSC-A) plot. (**C**) Lymphocyte subsets were identified using the markers CD4 and CD8 (CD4+ and CD8+ T cells). Fluorescent dyes used were PerCP for CD4 and Brilliant Violet 510 (BV510) for CD8. (**D**) Using specific fluorochrome-stained antibodies and a negative isotype control with a 1% threshold, the proportion of CCR6 expression on CD4+ T cells were identified.

Flow cytometric analyses were done within four hours of phlebotomy. We measured the white blood cell count using Sysmex KX-21NTM (Sysmex Corporation, Kobe, Japan). The white blood cell count was used to calculate the blood needed to obtain 1.0 × 10^6^ white blood cells. A 1% lysis buffer (BioLegend, San Diego, CA, USA) was used, and the sample was stored for 10 minutes at room temperature in the dark to lyse erythrocytes. Cells were washed in BD FACS Flow isotonic buffer (BD Bioscience, San Jose, CA, USA) and spun in a centrifuge for five minutes at 500*g*. The supernatant was decanted, and the washing/centrifuging process was repeated an additional two times. After these steps, cells were resuspended in isotonic buffer and monoclonal fluorescent antibodies were added. We used Brilliant Violet 510 CD8 IgG1 (301048; BioLegend), phycoerythrin/cyanine7 (Cy7) CXCR3 IgG1 (353720; BioLegend), peridinin-chlorophyll-protein (PerCP) CD4 IgG2a (FAB3791C; R&D Systems, Minneapolis, MN, USA), Fluorescein isothiocyanate (FITC) CCR5 IgG2b (R&D Systems, FAB182F), FITC CCR6 IgG2b (353412; BioLegend). The following isotype controls were used: Brilliant Violet 510 IgG1 (400172; BioLegend), Pe/Cy7 IgG1 (400126; BioLegend), PerCP IgG2a (IC003C; R&D Systems), FITC IgG2b (400310; BioLegend). After incubation for 20 minutes in the dark at room temperature, the stained cells were washed and resuspended in isotonic buffer. The cells were analyzed on BD FACS Canto II flow cytometer (BD Bioscience, San Jose, CA, USA) with a gating size of 100.000 singlet leukocytes. Flow data were analyzed with Kaluza Analysis software (v. 2.1; Beckman Coulter, Inc, Southfield, MI, USA). An example of the gating strategy is shown in Figure 1.

EDTA full blood from patients were used for genomic DNA extraction and SNP analyses. Genomic DNA extraction and SNP analyses were performed at BioXpedia, Denmark. SNP genotyping were analyzed by using the Fluidigm GT192.24 Dynamic Array Integrated Fluidic Circuit (Fluidigm Corp., South San Francisco, CA, USA) and performed according to the manufacturer’s protocol. Data were imported to the Fluidigm SNP Genotyping Analysis software v.4.5.1 and analyzed with standard settings. The following SNPs were analyzed: CFH rs1061170 and ARMS2 rs10490924.

### Statistical Analyses

All analyses were performed using R studio package 4.2.1. Normally distributed data are reported using mean and 95% confidence interval (CI), and non-normally distributed data presented using median and interquartile range. Group comparisons were made using independent samples t-test or Wilcoxon rank sum test. Normality was assessed with histograms and QQ-plots. Categorical data are compared using χ² test and are presented with numbers and percentages. Multiple regression analysis was used to assess the effect of possible confounding variables. Associations were assessed with linear regression. A Bland-Altman plot was used to demonstrate the level of agreement between the two methods: Heidelberg RegionFinder and the applied MATLAB-script. *P* values < 0.05 were interpreted as statistically significant. Power calculations were based on previous similar immunologic studies on AMD patients, a sample size of minimum 26 in each group is necessary to obtain a power of 80%.^17^^,^^45^

## Results

### Participants Characteristics

A total of 143 participants were included in the study, of which 13 participants were excluded post-hoc: four patients developed nAMD, three were diagnosed with cancer, three died, one had CRP levels above 16, and two participants were lost to follow-up. Thus 130 participants were included in the final analysis: 85 patients with GA and 45 were healthy control individuals. Patients with GA were subdivided into fast progressors (n = 28) and slow progressors (n = 29) based on the upper and lower square root PR tertiles. Participant characteristics are summarized in Table 1. Apart from differences in age between patients with GA and healthy controls, the groups did not differ significantly in gender, lifestyle, or comorbidities.

**Table 1.** Participant Characteristics

	Diagnosis				*P* Value	
	GA (n = 85)	sGA (n = 29)	fGA (n = 28)	HC (n = 45)	GA vs HC	sGA vs fGA
Age (y), mean (95%CI)	80.2 (79–82)	80.3 (78–83)	80.5 (78–84)	72.8 (71–75)	**<0.001** ^*^	0.941^*^
Sex						
** **Female	49 (58%)	17 (59%)	22 (79%)	20 (44%)	0.211^†^	0.303^†^
** **Male	36 (42%)	12 (41%)	6 (21%)	25 (56%)		
Body mass index, mean (95% CI)	26.5 (26–27)	26.6 (25–28)	26.0 (24–27)	27.4 (26–29)	0.254^*^	0.589^*^
Hypertension	55 (65%)	19 (66%)	19 (68%)	23 (51%)	0.19^†^	>0.99^†^
Hypercholesterolemia	29 (34%)	7 (24%)	8 (29%)	22 (49%)	0.15^†^	0.937^†^
Cardiovascular disease	38 (45%)	11 (38%)	11 (39%)	15 (33%)	0.29^†^	0.913^†^
Type 2 diabetes	8 (9%)	4 (14%)	1 (4%)	8 (18%)	0.27^†^	0.371^†^

HC, healthy controls; sGA, slow GA progressors; fGA, fast GA progressors.

*P* values in bold are significant.

* Two sample t-test.

† χ^2^ test.

### Progression of GA Lesion Area

**Figure 2.**
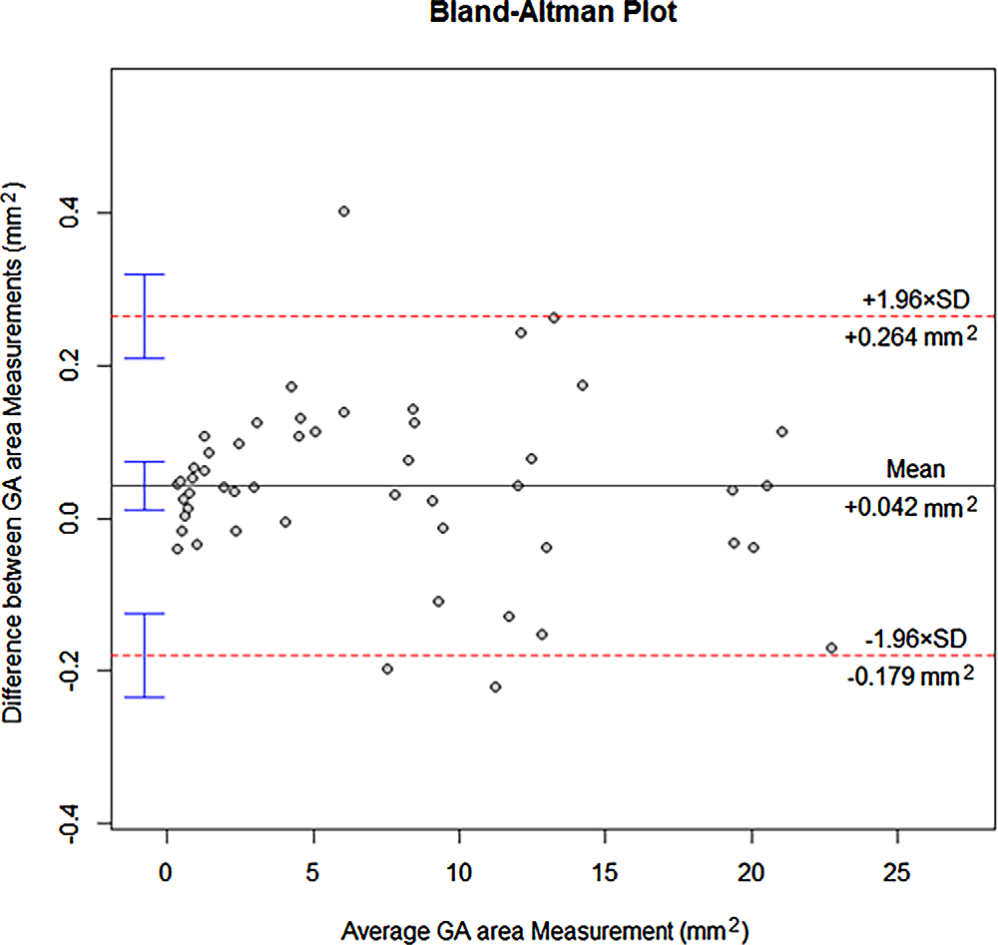
Comparison between methods using Heidelberg RegionFinder versus MATLAB-based software for measurements of GA areas. A Bland-Altman plot demonstrating differences in GA area measurements between methods using Heidelberg RegionFinder and MATLAB-based software. The x-axis displays the average GA area of the two methods being compared. The y-axis displays the difference in GA area between the two methods. Values above zero on the y-axis represent higher GA area values for the MATLAB-based software method than for Heidelberg RegionFinder-based software method. The *black solid line* represents the mean GA area difference between the two methods. Mean GA area difference was 0.042 mm^2^ (95% CI, 0.010-0.074). The *red dashed lines* represent the lower and upper limits of agreement, 0.264 mm^2^ (95% CI, 0.208 to 0.319) and −0.179 mm^2^ (95% CI, −0.235 to −0.124), respectively. *Vertical blue bars* represent the 95% CI.

The mean follow-up period was 13.1 months (range, 7–22 months). Inter-rater agreement of lesion area measurement between two graders had an interclass correlation coefficient of >0.99 (95% CI, 0.99-1), indicating an excellent agreement. Mean baseline area of atrophy was 6.71 mm² (95% CI, 5.27-8.15), and mean PR of the atrophic lesion was 1.45 mm²/y (95% CI, 1.15-1.74). These measured PR were similar to those previously described in studies of GA natural history.^46^^,^^47^ Following square root (sqrt) transformation slow GA progressors had a mean sqrt PR of 0.10 mm/y (95% CI, 0.08-0.11), and fast GA progressors a mean sqrt PR of 0.48 mm/y (95% CI, 0.44-0.53). The Bland-Altman difference plot for comparison of the two methods, Heidelberg RegionFinder and MATLAB-based software, showed a good agreement with a mean difference in GA area measurement of 0.042 mm^2^ (95% CI, 0.010-0.074) (Fig. 2).

### Chemokine Receptor Expression and Relation to Risk-Associated SNPs

#### CCR6

We found that GA patients had a significantly lower proportion of CCR6 on CD8+ T cells compared to healthy controls (median [interquartile range]: 4.04% [2.69%] vs. 6.00% [6.22%], *P* = 0.005). In the GA group, patients with fast GA progression had a lower percentage of CCR6 on CD4+ T cells (17.4% [8.59%]) compared to slow GA progressors (22.4% [12.5%], *P* = 0.019) (Table 2).

**Table 2.** Percentage of CD4+ and CD8+ T Cells Expressing Surface CXCR3, CCR5, and CCR6

	Diagnosis				*P* Value^*^	
	GA (n = 85)	sGA (n = 29)	fGA (n = 28)	HC (n = 45)	GA vs HC	sGA vs fGA
CD4+ T cells						
** **CXCR3+	1.79 (2.67)	1.41 (2.59)	1.72 (2.43)	2.61 (4.72)	0.054	0.917
** **CCR5+	0.41 (0.53)	0.38 (0.50)	0.45 (0.47)	0.25 (0.43)	**0.010**	0.744
** **CCR6	21.9 (14.6)	22.4 (12.5)	17.4 (8.59)	26.3 (14.4)	0.051	**0.019**
CD8+ T cells						
** **CXCR3+	4.00 (6.38)	4.01 (7.30)	3.06 (5.04)	6.47 (8.66)	**0.003**	0.582
** **CCR5+	11.2 (20.4)	11.8 (16.1)	8.63 (21.7)	6.37 (14.1)	**0.005**	0.817
** **CCR6	4.04 (2.69)	3.75 (3.56)	4.03 (2.29)	6.00 (6.22)	**0.005**	0.854

HC, healthy controls; sGA, slow GA progressors; fGA, fast GA progressors.

* Wilcoxon rank sum test. Measures are reported as median and interquartile range. *P* values in bold are significant.

#### CCR5

Patients with GA had an increased proportion of CCR5 on CD4+ and CD8+ T cells compared to healthy controls (0.41% [0.53%] vs. 0.25% [0.43%], *P* = 0.010 and 11.2% [20.4%] vs. 6.37% [14.1%], *P* = 0.005, respectively) (Table 2). After stratification of GA patients according to ARMS2 SNP, we found a significant lower proportion of CCR5 on CD8+ T cells in patients carrying high-risk TG and TT genotypes compared to GA patients with the low-risk GG genotype (8.66% [18.6%] vs. 18.9% [18.6%], *P* = 0.007) (Table 3). The investigated chemokine receptors were also stratified according to CFH SNP; however, we did not find them to reach statistical significance (results are not shown).

**Table 3.** Percentage of CXCR3, CCR5 and CCR6 on Lymphocyte Subsets Stratified According to ARMS2 rs10490924

	ARMS2 rs10490924		
	TG,TT Genotype (n = 55)	GG Genotype (n = 30)	*P* Value^*^ TG,TT vs GG Genotype
CD4+ T cells			
** **CCR5+	0.38 (0.57)	0.44 (0.46)	0.294
** **CCR6+	21.2 (14.9)	22.1 (13.2)	0.332
** **CXCR3+	1.52 (2.04)	2.53 (2.67)	0.069
CD8+ T cells			
** **CCR5+	8.66 (18.6)	18.9 (18.6)	**0.007**
** **CCR6+	4.36 (3.30)	3.65 (2.10)	0.113
** **CXCR3+	3.74 (6.41)	4.96 (5.76)	0.579

Measures are reported as median and interquartile range. *P* values in bold are significant.

* Wilcoxon rank sum test.

#### CXCR3

We found a significantly lower proportion of CXCR3 on CD8+ T cells in patients with GA compared to healthy controls (4.00% [6.38%] vs. 6.47% [8.66%], *P* = 0.003). Furthermore, we explored whether any difference in expression levels could be attributed to the age difference found between GA patients and healthy controls. Age was not associated with any of the investigated markers. We particularly investigated this considering previous studies finding CCR5 to be higher expressed in elderly compared to younger individuals.^9^^,^^10^ Multiple regression analysis showed that the differences observed between the groups, were not related to arterial hypertension, hypercholesterolemia, cardiovascular disease, or type 2 diabetes.

## Discussion

Halting progression of GA is pivotal for stabilizing visual function in GA. Our findings suggest a role for CCR6 in occurrence and progression rate in GA, making it a candidate molecule for further investigation.

The chemokine receptor CCR6 which binds specifically to CCL20, is expressed on dendritic cells, NK cells, B cells, CD4+ and CD8+ T cells.^48^^–^^51^ CCR6 has been associated with the pathogenesis of experimental autoimmune encephalitis and uveitis, psoriasis, asthma, and several other diseases.^52^^–^^55^ In response to CCL20 a positive amplification loop is initiated, in which Th17 cells migrate to target tissue to produce the proinflammatory cytokines IL-17A and IL-23, which further upregulates CCL20 expression and recruits more Th17 cells. Lymphocytes have been observed in the choroid of eyes with AMD^4^^–^^6^ and proinflammatory cytokines secreted by Th17 cells have been shown to be capable of breaching the blood-retinal barrier.^56^^,^^57^

In previous studies conducted by our group, CCR6 expression on CD4+ T cells was lower in patients with nonexudative AMD compared to healthy controls. However, the GA group was not studied as an individual group but as part of Clinical Age-Related Maculopathy Staging score 2–4 (nonexudative), where the majority of patients had intermediate AMD.^14^ Nonetheless, these findings are consistent with our results, reporting lower levels of CCR6 in GA patients, and that this phenomenon is associated with progression of disease. CCR6 has been shown to be a representative marker for Th17 cells.^58^^–^^61^ Thus a dysregulation of Th17-mediated immune response seems to occur in GA patients in general and specifically in patients with fast progression of GA. Intriguingly, CCR6 has also shown to be expressed by immunosuppressive regulatory T cells (Tregs),^62^^–^^64^ which are a subset of CD4+ T cells that are able to dampen effector T-cell immune responses. An alternative interpretation of our results could therefore be that patients with fast progression of disease have a lower immunosuppression, thereby fostering a proinflammatory milieu that could lead to inadvertent tissue damage. A dysfunctional immunoregulation in fast progressors caused by an imbalance of Tregs seems most plausible; however, this warrants further investigation.

The chemokine receptor CXCR3 is expressed on several cell types and has a wide variety of functions, such as regulation of leukocyte migration, T cell activation and differentiation.^65^^–^^67^ CXCR3 is activated by the binding of the IFN-ɣ–inducible chemokines CXCL9, CXCL10 and CXCL11, which have distinct roles in regulating T cell immune responses. CXCL9 and -10 are considered proinflammatory, whereas CXCL11, which binds CXCR3 with the highest affinity, activates a different signaling pathway restricting inflammation.^68^ In humans, three splicing variants of CXCR3 are found each activating different intracellular signaling pathways. The binding of different chemokines to a chemokine receptor, activating different pathways can result in distinct outcomes, a phenomenon known as “biased agonism” or “biased signaling.” These biased responses can be altered by the type of chemokine ligand, the receptor variant, and the target tissue or cell type,^68^^–^^70^ further adding complexity to the chemokine receptor axis.

The role of CXCR3 has mainly been studied in relation to nAMD, with emphasis on its antiangiogenic properties.^13^ The underlying molecular mechanism driving this angiostatic function is, however, not fully understood. CXCR3 is expressed on choroidal endothelial cells^71^ and binding of its ligand CXCL10 have shown to reduce the migration of endothelial cells and their ability to form tubes.^24^ Another study demonstrated that CXCR3-deficient mice developed larger size choroidal neovascularization with more fluid leakage and macrophage-infiltration into choroidal neovascularization affected areas compared to wild-type mice.^13^ In line with this, our group and others have found a lower expression of CXCR3 on peripheral T cells in patients with nAMD.^14^^,^^16^^,^^22^

In this study, levels of CXCR3 on T cells were shown to be lower in GA patients compared to healthy controls. Interestingly, our group did not previously find a significant difference in CXCR3 expression in GA patients.^16^^,^^18^ The discrepancy in the results could be attributed to the lower number of GA patients included in our past study. Due to the complexity of the CXCR3 axis, we can only speculate on how the mechanism of activated signaling pathways in target cells in GA patients behave compared to those in patients with nAMD. Further studies investigating the role of CXCR3 in an in vitro and in vivo setting, are needed to determine the distinct signaling pathways induced by CXCR3.

The chemokine receptor CCR5 is expressed on T cells, NK cells, and dendritic cells, among others, and is activated on binding to its chemokine ligands CCL3, CCL4 and CCL5, to mention a few. In a murine study, Duncan et al.^72^ found CCR5 and CCL5 to be expressed in the inner retina. Furthermore, CCL5 was shown to be secreted from both the apical and basolateral side of human RPE cells suggesting that the basolateral secretion could influence peripheral leukocytes.^73^ Only a few studies have investigated the peripheral expression of CCR5 and CCL5 in AMD patients. However, CCR5 and CCL5 have been studied in relation to the pathophysiology of other age-related neurodegenerative diseases such as Parkinson's and Alzheimer's disease. Evidence points to CCR5-CCL5 interaction having a neuroprotective role. For instance, a study observed loss of dopaminergic neurons in the brain of CCR5-deficient mice in comparison to wild-type mice^74^ and CCL5 promoted neuronal survival under proapoptotic conditions.^75^

In our study we found an increased proportion of CCR5 in patients with GA, which is in accordance with our previous findings.^18^ Interestingly, we also found that patients with high-risk genotypes of ARMS2 polymorphism had a lower proportion of CCR5 on CD8+ T cells compared to patients carrying the low-risk genotype. Considering the proposed neuroprotective role of CCR5, our findings could suggest that patients with genetical predisposition have some degree of neuroprotection loss.

Important limitations should be considered when interpreting our results. First, the study is observational in its character, and we can only speculate on causality. Second, patients were followed up after a minimum seven months (range 7–22). It can be argued if this seems too short for evaluating the progression of GA. However, only three of the 85 patients were followed up after seven to nine months with the remaining patients completing follow-up examination between nine to 22 months. Last, levels of the chemokine ligands corresponding to the investigated chemokine receptors were not measured. This could have given us further insight into the ligand-chemokine receptor interaction, thereby providing us with a deeper understanding of the pathogenesis of GA.

In conclusion, our results demonstrate a peripheral dysregulation of chemokine receptors in GA patients. Genotypic risk stratification of our GA cohort further underlines the differences in said levels between subgroups of GA. To our knowledge, stratification of GA patients based on risk-associated SNPs and progression rate in relation to chemokine receptor levels has not previously been studied. The immune responses triggered by chemokines and chemokine receptors on the RPE level in GA patients is not fully understood. Further studies are needed to uncover chemokines and chemokine receptors of potential clinical relevance to identify novel target molecules for the treatment of GA.
